# Mast Cell Coupling to the Kallikrein–Kinin System Fuels Intracardiac Parasitism and Worsens Heart Pathology in Experimental Chagas Disease

**DOI:** 10.3389/fimmu.2017.00840

**Published:** 2017-08-02

**Authors:** Clarissa R. Nascimento, Daniele Andrade, Carla Eponina Carvalho-Pinto, Rafaela Rangel Serra, Lucas Vellasco, Guilherme Brasil, Erivan Schnaider Ramos-Junior, Julia Barbalho da Mota, Larissa Nogueira Almeida, Marcus V. Andrade, Maria de Nazaré Correia Soeiro, Luiz Juliano, Patrícia Hessab Alvarenga, Ana Carolina Oliveira, Fernando Lencastre Sicuro, Antônio C. Campos de Carvalho, Erik Svensjö, Julio Scharfstein

**Affiliations:** ^1^Instituto de Biofísica Carlos Chagas Filho, Universidade Federal do Rio de Janeiro (UFRJ), Rio de Janeiro, Brazil; ^2^Departamento de Imunobiologia, Universidade Federal Fluminense (UFF), Niterói, Brazil; ^3^University of the Pacific, San Francisco, CA, United States; ^4^Faculdade de Medicina, Universidade Federal de Minas Gerais (UFMG), Belo Horizonte, Brazil; ^5^Departamento de Clinica Medica, Universidade Federal de Minas Gerais (UFMG), Belo Horizonte, Brazil; ^6^Instituto Oswaldo Cruz, Fundação Oswaldo Cruz (Fiocruz), Rio de Janeiro, Brazil; ^7^Universidade Federal de São Paulo (UNIFESP), São Paulo, Brazil; ^8^Instituto de Bioquímica Médica Leopoldo de Meis (IBqM), Universidade Federal do Rio de Janeiro, Rio de Janeiro, Brazil; ^9^Universidade do Estado do Rio de Janeiro (UERJ), Centro Biomédico Rio de Janeiro, Rio de Janeiro, Brazil

**Keywords:** bradykinin, Chagas disease, endothelin, G protein-coupled receptors, kallikrein, mast cells, *Trypanosoma cruzi*

## Abstract

During the course of Chagas disease, infectious forms of *Trypanosoma cruzi* are occasionally liberated from parasitized heart cells. Studies performed with tissue culture trypomastigotes (TCTs, Dm28c strain) demonstrated that these parasites evoke neutrophil/CXCR2-dependent microvascular leakage by activating innate sentinel cells *via* toll-like receptor 2 (TLR2). Upon plasma extravasation, proteolytically derived kinins and C5a stimulate immunoprotective Th1 responses *via* cross-talk between bradykinin B2 receptors (B2Rs) and C5aR. Awareness that TCTs invade cardiovascular cells *in vitro via* interdependent activation of B2R and endothelin receptors [endothelin A receptor (ET_A_R)/endothelin B receptor (ET_B_R)] led us to hypothesize that *T. cruzi* might reciprocally benefit from the formation of infection-associated edema *via* activation of kallikrein–kinin system (KKS). Using intravital microscopy, here we first examined the functional interplay between mast cells (MCs) and the KKS by topically exposing the hamster cheek pouch (HCP) tissues to dextran sulfate (DXS), a potent “contact” activator of the KKS. Surprisingly, although DXS was inert for at least 30 min, a subtle MC-driven leakage resulted in factor XII (FXII)-dependent activation of the KKS, which then amplified inflammation *via* generation of bradykinin (BK). Guided by this mechanistic insight, we next exposed TCTs to “leaky” HCP—forged by low dose histamine application—and found that the proinflammatory phenotype of TCTs was boosted by BK generated *via* the MC/KKS pathway. Measurements of footpad edema in MC-deficient mice linked TCT-evoked inflammation to MC degranulation (upstream) and FXII-mediated generation of BK (downstream). We then inoculated TCTs intracardiacally in mice and found a striking decrease of parasite DNA (quantitative polymerase chain reaction; 3 d.p.i.) in the heart of MC-deficient mutant mice. Moreover, the intracardiac parasite load was significantly reduced in WT mice pretreated with (i) cromoglycate (MC stabilizer) (ii) infestin-4, a specific inhibitor of FXIIa (iii) HOE-140 (specific antagonist of B2R), and (iv) bosentan, a non-selective antagonist of ET_A_R/ET_B_R. Notably, histopathology of heart tissues from mice pretreated with these G protein-coupled receptors blockers revealed that myocarditis and heart fibrosis (30 d.p.i.) was markedly and redundantly attenuated. Collectively, our study suggests that inflammatory edema propagated *via* activation of the MC/KKS pathway fuels intracardiac parasitism by generating infection-stimulatory peptides (BK and endothelins) in the edematous heart tissues.

## Introduction

Although the complement system is a classic example of a proteolytic cascade that stimulates immunity through the generation of proinflammatory peptides, studies in different infectious disease models extended this concept to the kallikrein–kinin system (KKS) (1). Intravascular activation of the KKS is initiated when the zymogen factor XII (FXII) interacts with a broad range of endogenous “contact” substances (2), including negatively charged polymers, such as platelet-derived polyphosphates (polyP) (3) and DNA associated with neutrophil extracellular traps (4). Trace formation of FXIIa in the blood is sufficient to convert the prekallikrein into active plasma kallikrein (PKa), a serine protease that reciprocally cleaves FXII. Feedback cycles of activation between PK/FXII lead to FXIIa-mediated cleavage of FXIa. Further downstream, FXIa generates FIXa—the effector of fibrin formation *via* the intrinsic pathway of coagulation. In parallel, PK activates the proinflammatory KKS by proteolytically excising bradykinin (BK) from an internal moiety of high molecular weight kininogen (HK). Acting as a paracrine mediator, the short-lived BK induces vasodilation and increases microvascular permeability *via* activation of endothelial bradykinin B2 receptor (B2R), a constitutively expressed subtype of kinin receptor (5). In addition, PK promotes plasmin-dependent fibrinolysis and C3 activation, hence couples FXIIa-dependent thrombogenesis to fibrinolytic and immunological mechanisms. As inflammation persists, a GPI-linked carboxypeptidase M removes the C-terminal arginine from the primary kinin, thus converting the B2R agonist into a high-affinity ligand for bradykinin B1 receptor (B1R) (6), a G protein-coupled receptor (GPCR) subtype that is transcriptionally upregulated in injured tissues by proinflammatory cues, such as IL-1β, TNF-α (7, 8), or by prooxidative polypeptides, e.g., angiotensin II and endothelin-1 (ET-1) (9).

During infection or sterile inflammation, subtle increases in endothelial permeability allow for the extravascular accumulation/diffusion of plasma proteins, including complement components and blood-borne kininogens (high or low molecular weight) (1). Using animal models of severe MC-mediated allergic reactions, Sala-Cunill et al. (10) showed that BK fueled inflammation via mechanisms involving activation of FXII by endogenous contact factors released from MC granules, e.g., heparin (11) and/or PolyP (12). Once liberated by PK, the short-lived BK rapidly potentiates allergic inflammation *via* iterative cycles of B2R-dependent activation of the endothelium and plasma leakage. During infection, kinins can be released extravascularly by the action of microbial kininogenases, such as cruzipain and gingipains, i.e., cysteine proteases respectively expressed by the parasitic protozoan *Trypanosoma cruzi* (13) and the periodontal bacteria *Porphyromonas gingivalis* (14, 15), both of which object of systematic investigations in vitro and in vivo.

Afflicting approximately 8 million people in Latin America (16), Chagas disease, the heart pathology caused by chronic *T. cruzi* infection, remains incurable (17). Human infection starts when the blood seeking triatomine releases infective forms (metacyclic trypomastigotes) on tissues lacerated by the proboscis. Alternatively, *T. cruzi* is transmitted orally following ingestion of macerated fruit juices that were contaminated with infected bugs (18, 19). The life cycle of *T. cruzi* in mammals requires an obligate stage of intracellular development in a broad range of host cells, including epithelial cells, tissue-resident macrophages, and cardiomyocytes. After invading host cells *via* a mechanism akin to endocytosis, the trypomastigotes rapidly escape from parasitophorous vacuole and reach the host cell cytoplasm, where they transform into oval-shaped amastigotes (replicating forms). After coopting the host cell metabolism to support amastigote division for several days (20), the amastigotes transform into infective trypomastigotes. Upon cell death, the extracellular trypomastigotes navigate away from the primary foci of infection and enter the bloodstream from where the infection is disseminated. Characterized by high blood parasitemia and systemic inflammation, acute Chagas disease subsides with the onset of adaptive immunity. However, a low-grade infection (asymptomatic) persists for several years before development of clinical symptoms. Although a minority of patients present digestive system complications (megacolon), about 25% of the chronically infected chagasic patients develop a progressive and as yet incurable cardiomyopathy [chronic chagasic cardiomyopathy (CCC)] (21).

Clinical and histopathological studies in *T. cruzi*-infected mice have linked severity of CCC to immunoregulatory abnormalities (22). Systematic investigations in mice models of chronic Chagas disease indicated that chronic myocarditis/fibrosis worsens as a consequence of an imbalance between protective versus pathogenic subsets of heart-infiltrating IFN-γ and perforin-producing CD8^+^ T cells (23). More recently, cohort studies performed in Spain revealed that chronic chagasic patients display a hyper-coagulopathy phenotype (24). Although the role of the contact system was not explored in this particular study, these findings raise the possibility that, over the years, activated monocytes circulating in the bloodstream of chronic chagasic patients might forge the coupling between the extrinsic/intrinsic pathways of coagulation (1). While not excluding a primary role for infection-associated immunopathology (25), it was reported that patients with chronic myocardiopathy display marked abnormalities in heart microvessels and extracellular matrix. Insight on the molecular basis of CCC emerged from experimental studies (26) showing that parasitized cardiomyocytes upregulate the expression of ET-1, a potent vasoconstrictor and profibrosing polypeptide. Extending this analysis to *in vivo* setting, these authors showed evidence that heart fibrosis was attenuated in transgenic mice in which ET-1 was genetically ablated from cardiomyocytes—but not from endothelial cells. While these studies were in progress, our group suggested that generation of kinins may have a dual role in host/parasite equilibrium (27). Acting on the behalf of host defenses, the released kinins stimulate B2R expressed by immature dendritic cells (28), hence convert these antigen-presenting cells into inducers of IFN-γ-producing (immunoprotective) effector CD4 and CD8 T cells (29–31). Although limited to *in vitro* studies, the hypothesis that *T. cruzi* might reciprocally benefit from the activation of the KKS (27) was initially supported by evidence that tissue culture trypomastigotes (TCTs) (Dm28c strain) invade non-professional phagocytic cells (e.g., smooth muscle cells and cardiomyocytes) *via* cross-talk between B2R, B1R and endothelin receptors [endothelin A receptor (ET_A_R)/endothelin B receptor (ET_B_R)] (13, 27, 32, 33). Using multiple infection models, here we demonstrate that inflammatory edema propagated *via* the MC/KKS pathway fuels heart parasitism and worsens myocarditis/fibrosis by generating infection-promoting peptides, such as kinins and endothelins. Our study illustrates how endothelial barrier destabilization, a common manifestation of inflammation, might provide *T. cruzi* with an opportunity to rapidly invade surrounding host cells, hence ultimately escaping from humoral immunity.

## Materials and Methods

### Ethics Statement

All animal care and experimental procedures were performed in accordance to the Brazilian guidelines (Brazilian Directive for Care and Use of animals for Teaching and Research—DBCA) published by the Brazilian Council for Control of Animal Experimentation (Conselho Nacional de Controle de Experimentação Animal—CONCEA, http://www.mct.gov.br/upd_blob/0234/234054.pdf) and Federal Law 11.794 (October 8, 2008). Studies involving animals are reported in compliance with the ARRIVE guidelines (34, 35). The protocols used in this study were approved by the Institutional Ethical Committee of Federal University of Rio de Janeiro (code number: IBCCF 101, and license protocol number 014).

### Mice Inbred Strains

Colonies of C57BL/6 and BALB/c were purchased from our facilities (UFRJ, Rio de Janeiro). B6-Kit^+/+^ (wild type) and B6-KitW-sh/W-sh (MC-deficient) were provided by one of the co-authors (M.A.; UFMG, Belo Horizonte). The experiments were conducted with male or female mice, 8- to 12-weeks-old, weighting 20–25 g and housed in ventilated cages under specific pathogen-free conditions, in our animal facilities at 22 ± 2°C with a 12 h light dark cycle, with access to food (normal diet) and water *ad libitum*. Mice were randomly allocated to each experimental group.

### Antibodies and Reagents

FITC–dextran 150 kDa and dextran sulfate (DXS) 500 kDa were purchased from TdB Consultancy (Uppsala, Sweden). Heparin 4 kDa (enoxaparin EUROFARMA Versa, NC 154693) was kindly provided by PAS Mourão (Universidade Federal do Rio de Janeiro). HOE-140, B2R antagonist (B2RA) was purchased from Sigma (St. Louis, MO, USA). Bosentan was a donation from Actelion (Basel, Switzerland). FXII inhibitor (36) was a generous gift from Dr. Thomas Renné (Karolinska Institute). Cromoglycate, mepyramine, histamine, captopril were purchase from Sigma (St. Louis, MO, USA). PKSI-527 and PdSP15a were supplied by the co-authors (LJ) and (PA), respectively.

### Generation of TCTs

Tissue culture trypomastigotes (Dm28c, originally a gift from Dr. Samuel Goldenberg, Instituto Carlos Chagas, Fiocruz, Parana) were harvested from the supernatants of cultures of monkey kidney fibroblast cell line LLC-MK2 (American Type Culture Collection—CCL-7) 5 days after infection. The cultures were maintained in Dulbecco’s Modified Eagle Medium (DMEM), 2% heat inactivated fetal calf serum (FCS). The freshly released TCTs were washed twice with excess Hank’s Balanced Saline Solution (HBSS) before being tested *in vitro* or *in vivo*, as described below.

### Cell Culture

Mouse heart cells were isolated in the laboratory of one of the co-authors (MS) as previously described (37) and seeded on coverslips laid in 24-well plates. Primary cultures of mice embryonic heart cells were maintained at 37°C in 5% CO_2_ atmosphere in DMEM, 10% FCS to get synchronized contractility.

### Invasion Assays

Tissue culture trypomastigotes were added to monolayers of primary heart cells (parasite ratio of 1:1) in DMEM containing 1 mg/ml human serum albumin (HAS—Baxter Pharmaceutical, Deerfield, IL, USA). Where indicated, the DMEM-HSA medium was supplemented with the angiotensin-converting enzyme (ACE) inhibitor lisinopril (25 µM) and/or the following compounds (isolated or combined): HOE-140 (B2RA) (0.1 µM), or cromoglycate (140 µg/ml). Stock solutions of HOE-140 were prepared in apyrogenic water and cromoglycate in PBS. Optimal concentration of HOE-140 was defined based on dose–response curves reported elsewhere (33). Host/parasite interactions proceeded for 3 h at 37°C in a humidified incubator, in a 5% CO_2_ atmosphere. At the end point of the assay, the infected monolayers were washed three times with HBSS, fixed in Bouin and staining with Giemsa. The infection was determined by counting the number of intracellular parasites in a total of 100 cells per coverslip. Assays were performed in triplicates.

### Paw Edema Measurements

B6-Kit^+/+^, B6-kitW-sh/W-sh, and BALB/c mice, under ketamine and xilazyne anesthesia (80 and 8 mg/kg, respectively), were injected into the hind footpad with Dm28c TCTs (10^6^ parasites in 10 µl of PBS) or with PBS (contralateral paw as a control). The sequential measurement of test and contralateral paws allows each mouse to serve as its own control, thus reducing animal use. Altogether, 165 mice were used. These assays were performed as previously described (29, 31, 38). Where indicated, animals were pretreated (−1 h) with a single-dose of ACE inhibitor captopril (4 mg/kg, i.p.) to minimize the degradation of kinins; HOE-140 (100 µg/kg—s.c.); cromoglycate (20 mg/kg—i.v.); histamine H1 receptor (H1R) antagonist mepyramine (10 mg/kg—i.p.), or infestin-4 (FXII inhibitor; 4.5 mg/kg—i.v.). Paw volume (3 h p.i. or 24 h p.i.) was measured with the aid of a plethysmometer. In the interval between measurements of 3 and 24 h, animals were housed following the conditions described above (Mice inbred strains). At the end of the experiments, mice were euthanized by CO_2_ inhalation. The data obtained were expressed as difference in volume (microliters) between the test and control paws.

### Microvascular Permeability Measurements—Intravital Digital Microscopy

Hamsters were purchased from Anilab (São Paulo, Brazil) and maintained in our animal facilities. The experiments were conducted with male hamsters, 3 months old, weighting 110–120 g. Altogether, 134 hamsters were used and the procedures were approved by the local ethical committee (IBCCF, license protocol number 014). Animals were anesthetized by injection of sodium pentobarbital (i.p.) supplemented with α-chloralose (2.5% W/V, solution in saline, i.v.) through a femoral vein catheter as previously described (39, 40). During the experimental procedures anesthesia was monitored by reflex measurement and supplemented with α-chloralose (2.5% W/V, solution in saline, i.v.) through a femoral vein catheter whenever required. A tracheal cannula (PE 190) was inserted to facilitate spontaneous breathing and the body temperature was maintained at 37°C by a heating pad monitored with a rectal thermistor. The hamster cheek pouch (HCP) was prepared for intravital microscopy (IVM) as reported (14, 39). The microcirculation of the HCP was observed after intravenous injection of the tracer FITC-dextran 150 kDa (100 mg/kg) using an Axioskop 40 microscope, objective 4× and oculars 10× (Carl Zeiss, Germany), equipped with appropriate filter (490/520 nm) for observations of fluorescence in epiluminescence (Colibri 2, Carl Zeiss, Germany). HCPs were continuously superfused with a HEPES-bicarbonate-buffered saline solution (pH 7.4; composition in mM: 110.0 NaCl, 4.7 KCl, 2.0 CaCl_2_, 1.2 MgSO_4_, 18.0 NaHCO_3_, 15.39 HEPES, and 14.61 Na HEPES) at a constant rate of 5 ml/min. A digital camera, AxioCamHRc, and a computer with the AxioVision 4.4 software program (Carl Zeiss) were used for image analysis of arteriolar diameter and total fluorescence in a representative rectangular area (5 mm^2^) of the HCP. The recorded fluorescence at 30 min after FITC-dextran injection [relative fluorescence units (RFUs)] in each experiment was adjusted to 2,000 RFU for statistical reasons. After an initial 30 min control period of continuous superfusion to verify normal microvascular flow in all vessels and absence of plasma leakage, HCPs were subjected to topical applications of different combinations of treatments, either added to superfusion solution or during interrupted superfusion in 500 µl of saline on top of the HCP-tissue. After the periods of interrupted superfusion, the flow was restarted. In a first group of experiments, HCPs were exposed to DXS 500 kDa (DXS—0.4, 2, or 4 µM) followed, or not, by application of histamine (4 µM), or by application of heparinized (heparin 4 kDa) hamster plasma (50 µl). In the second protocol, the HCP (steady state) was superfused for 10 min with captopril (10 µM) solution prior to the topical application of TCTs (3 × 10^7^) in the presence or absence of histamine (4 µM). At 60 min after topical application of TCTs, the HCPs were stimulated with histamine for 5 min as an internal control of normal reactivity of the microvasculature. Pharmacological interventions were carried out with cromoglycate (MC stabilizer, 40 mg/kg, i.p.), captopril (ACE inhibitor, 10 µM), HOE-140 (B2RA; 0.5 µM), PKSI-527 (PKa inhibitor, 20 µM) (41), and PdSP15a (contact phase inhibitor, 20 µM) (42). Dose–response experiments involved superfusion of HCPs for 10 min with captopril (10 µM) solution prior to the topical application of 60, 600, 6,000, and 60,000 TCTs/μl in 500 µl together with histamine 4 µM that were followed by histamine alone (4 µM) for 5 min at 60 min after TCT-application. At the end of each experiment, animals were euthanized by an overdose of pentobarbital and an i.v. injection of KCl 3 M.

### Contact System Activation

The activation of FXII/PK in citrated hamster plasma-treated (or not) with DXS 500 kDa was monitored by spectrofluorimetry as previously described (42, 43) using internally quenched fluorescent substrates whose sequences correspond to the C-terminal (Abz-GFSPFRAPRVQ-EDDnp) flanking region of BK of rat kininogen. The reaction was carried out in PBS, pH 7.4, using citrated hamster plasma (5%, v/v) or heparin (4 kDa), 4 µM of the Abz-peptidyl-EDDnp substrate, and 4 or 20 nM of the contact system activator DXS (500 kDa), at 37°C. The synthetic PKa inhibitor (PKSI-527, 10 µM) (41) was preincubated with hamster plasma for 5 min, at 37°C, prior to the addition of DXS and the substrate. The hydrolysis of the cleaved substrate Abz-peptidyl-EDDnp (Abz = *O*-aminobenzoyl and EDDnP = ethylenediamine 2,4-dinitrophenyl) was monitored by measuring the fluorescence at λex = 320 nm and λem = 420 nm in a fluorescence spectrophotometer (Spectramax M5–Molecular Device). Citrated or heparinized plasma (platelet free) was prepared by centrifugation of blood samples at 2,500 × *g* for 20 min at 4°C and quickly stored at −80°C in polypropylene tubes before use. After centrifugation, plasma samples were filtered using a 0.2 µm membrane.

### Echo-Guided Intracardiac Injection

Mice were trichotomized using a commercial depilatory gel. The animals were positioned at a holder that has conductive plates, enabling echocardiogram registration on the chest covered with ultrasound gel by means of a VisualSonic VEVO device 770 with 30-MHz transducer. The procedure was done under anesthesia provided by an isoflurane gas outlet while maintaining the temperature at 40°C. Heart was visualized in cross section in height of the papillary muscles lines and a needle was aligned with the transducer to display it near the left ventricular cavity. The aligned needle penetrated the myocardium, and after aspiration to verify if the cavity was accessed, we injected the suspension of Dm28c TCTs (10^6^ parasites) or PBS solution (volume of 50 µl) in the myocardium [lateral wall of the left ventricle (LV)]. After being removed from the holder, the animals were left in their cages where they recover from anesthesia within 5 min. Furthermore, animals were housed following the conditions described above (Mice inbred strains). At the end of the study, mice were euthanized by CO_2_ inhalation followed by the assessment of heart parasite load and histological analyses. Altogether, 180 mice were used.

### Histopathological Analyzes of Heart Tissues

The heart tissues from C57BL/6 mice infected as described above were isolated at 30 d.p.i and processed for evaluation of leukocyte infiltrates, MCs, and collagen deposition. Hearts were removed, washed, and softly squeezed in PBS until expel the blood before being fixed in formalin 5% for 24 h at room temperature. Hearts (cutting in half) were transferred to the tissue cassette and dehydrated through serial EtOH incubation for 30 min. They were then clarified in xylene (three times) for 30 min each. After 1 h in Erv-plast paraffin (Easypath-Brazil) at 58°C, hearts were embedded in the same paraffin. Serial sections (4 µm) were cut and after removal of the paraffin, they were incubated in serial EtOH followed by staining with H&E, Safranin, or Picrosirius Red, to evaluate respectively infiltrating leukocytes, MCs, and collagen fibers. Sections were visualized using a LEICA scan microscope with 20× objective (H&E, Safranin), and 20× or 10× objective (Picrosirius Red). Hearts from non-infected mice (PBS injected) were always included as controls and did not exhibit pathologic changes. Leukocyte infiltration was quantified by counting (±200 cells in 50 fields) in the entire pericardium area and myocardium near pericardium, where they were preferentially localized. Cardiac MCs were quantified by counting Safranin positive cells in the pericardium and near the pericardium area. For collagen quantification, six sections of one of the halves were entirely photographed. Using Image Pro-Plus (Image Processing and Analysis Software), we measured each photographed section and adjust as total area. The percentage of collagen area was calculated in relation to the total area.

### Measurements of *T. cruzi* DNA in the Mouse Tissues

*Trypanosoma cruzi* quantification was performed as previously described by our group (30) and others (44). C57BL/6, B6-Kit^+/+^, and B6-kitW-sh/W-sh mice were infected according to the description above. Where indicated, the animals were treated 1 h prior to infection with a single-dose of: (i) cromoglycate (20 mg/kg—i.v.), (ii) B2R antagonist HOE-140 (100 µg/kg—s.c.), (iii) ET_A_R/ET_B_R double antagonist Bosentan (30 mg/kg—i.p.) (45), or (iv) FXII inhibitor (4.5 mg/kg—i.v.) (36). Hearts were isolated 3 d.p.i., washed, softly squeezed in PBS until expel the blood and minced with surgical blades before DNA was isolated using the DNeasy blood and tissue Kit (QIAGEN) or PureLink genomic DNA mini Kit (Invitrogen, Carlsbad, CA, USA) according to the manufacturer’s instructions. Briefly, up to 25 mg of tissue was added to a 2 ml sample tube with lysis Buffer and proteinase K, mixed by gentle vortex and, subsequently, incubated at 56°C for 3 h. The DNA concentration was determined using the NanoDrop ND-1000 spectrophotometer (NanoDrop Technologies). The 260/280 ratio was calculated by spectrophotometer and used to evaluate the DNA purity (range between 1.8 and 2.0 was considered acceptable). Quantitative polymerase chain reaction (qPCR) for parasite quantification was performed using 100 ng of total DNA using a *T. cruzi* DNA dilution (standard curve). The standards for the qPCRs were generated using DNA from *T. cruzi* (Dm28c) epimastigotes. Considering that one parasite contains about 100 fg of DNA (46), we designed a curve with initial 2.5 ng (equivalent to 25,000 parasites with *C*_t_-mean 19) and serially diluted (1:10) until the lower limit of 2.5 fg (0.025 parasites with a *C*_t_-mean 37). The reactions were performed in a StepOne sequence detection system (Applied Biosystems, Warrington, UK) using SYBR Green PCR Master Mix (Applied Biosystems, Warrington, UK). The standard PCR conditions were 95°C (10 min), and then 45 cycles of 95°C (30 seg), 60°C (1 min), followed by the standard denaturation curve. Each reaction contained DNA template and 0.4 µM of each *T. cruzi*-specific primers GCTCTTGCCCACACGGGTGC (forward) and CCAAGCAGCGGATAGTTCAGG (reverse); and 0.1 µM of each primer for genomic B2m, CTGAGCTCTGTTTTCGTCTG (forward) and TATCAGTCTCAGTGGGGGTG (reverse). For quantification, *T. cruzi* measurement (average of triplicates) was corrected using the murine B2M-product (loading control, average of triplicates).

### Statistical Analysis

Data are presented as mean ± SD, unless otherwise indicated. The Student *t*-test and/or analysis of variance (ANOVA and Wilcoxon) were used to determine statistical significance. When the mean values of the groups showed a significant difference, pair-wise comparison was performed using the Bonferroni test. Statistical significance was set at *P* < 0.05. All statistical analysis was performed with Prism 5.0 (GraphPad Software).

## Results

### Histamine and BK Potentiate the Proinflammatory Phenotype of TCTs

We have previously demonstrated that TCTs elicit plasma leakage *via* cooperation between tGPI, a mucin-linked phosphatidylinositol anchor that activates toll-like receptor 2 (TLR2) (47, 48) and cruzipain, a cysteine protease that releases kinins from surface bound HK and C5a from native C5 (31). More recently, studies of the pathogenesis of anaphylaxis linked severe MC-mediated reactions to BK release *via* heparin-induced activation of the contact system (10). Given this precedent, and limited information about the pathogenic role of MCs in Chagas disease (49, 50), we asked whether *T. cruzi*-induced inflammation was propagated *via* activation of the MC/KKS pathway. IVM in the HCP seemed to be an appropriate system to address this question because (i) MCs are predominantly positioned along arterioles of the HCP and respond to topically applied stimuli such as 48/80 (51), (ii) the pathogens are topically applied to the HCP (33), hence dispensing the use of needles—a procedure that causes bleeding and spurious KKS activation, and (iii) hamsters are susceptible to *T. cruzi* infection and exhibit a dilated chronic cardiomyopathy that closely resembles the human heart disease (52).

**Figure 1:**
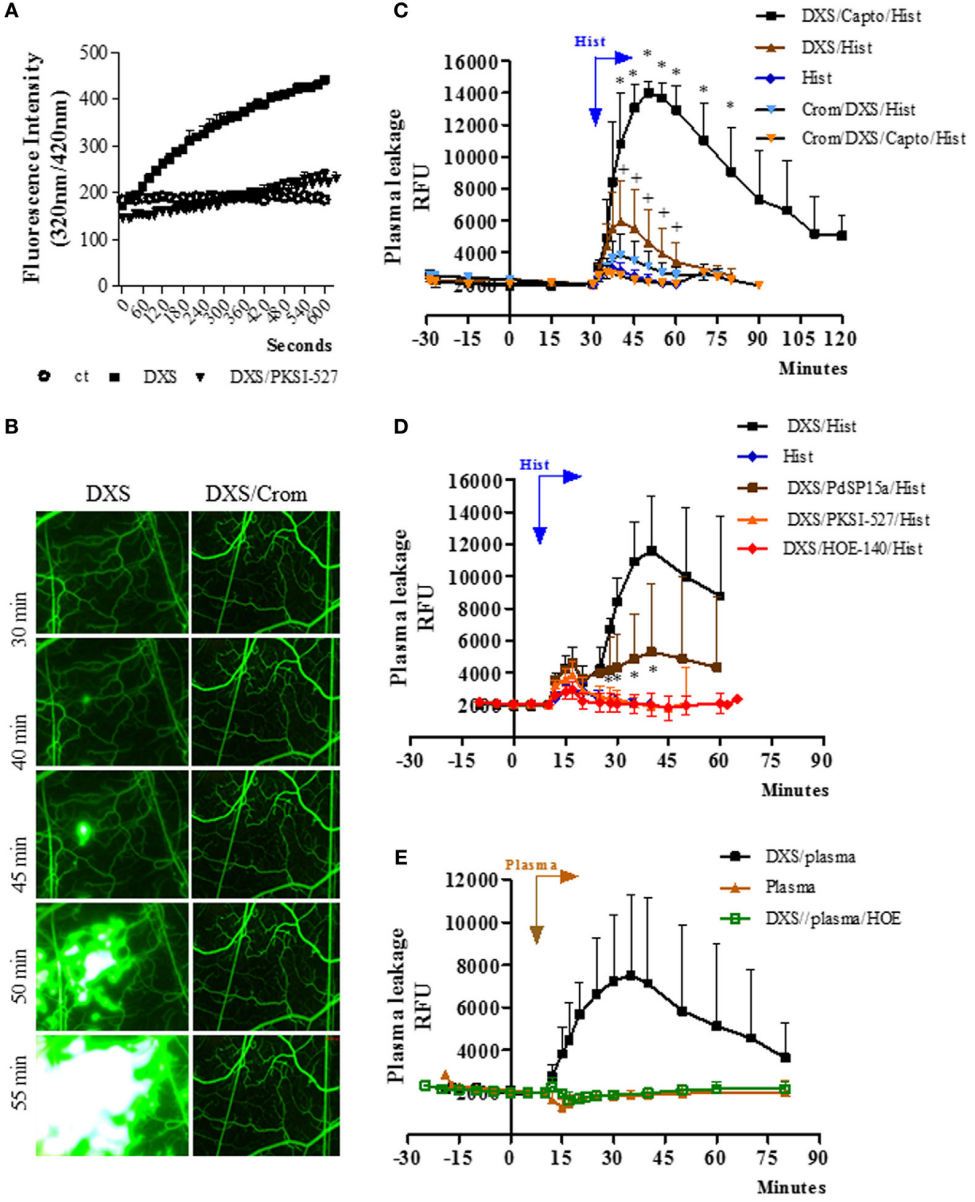
Bradykinin-induced microvascular leakage is propagated *via* coupling of mast cell and contact phase factors. **(A)** Enzyme assays performed with high molecular weight kininogen mimetic substrate detect plasma kallikrein (PKa) activity in fresh hamster plasma (citrated) incubated with the contact activator dextran sulfate (DXS) (20 nM). The kinetics of hydrolysis was performed in the presence/absence of the PKa inhibitor PKSI-527. Substrate hydrolysis was monitored by the increase of fluorescence with time (mean ± SD). Data are representative of three independent experiments run in duplicates. **(B–E)** Microvascular leakage in the hamster cheek pouch (HCP) sensitized with DXS. **(B)** HCPs were superfused with DXS (4 µM) for 45 min and fluorescence was visualized at different time-points (representative images, *n* = 6). Where indicated, the hamsters were pretreated with cromoglycate. **(C)** Synergism between histamine and DXS was studied after 30 min tissue superfusion, with medium supplemented with DXS 0.4 µM, in the presence or absence of captopril, followed by 5 min of histamine application, i.e., without any interruption of superfusion., DXS/Hist (*n* = 5), DXS/Capto/Hist (*n* = 5). Where indicated, the hamsters were pretreated i.p. with cromoglycate (*n* = 2, and *n* = 4, in the presence and absence of captopril, respectively). Histamine-induced leakage (*n* = 12) was inserted (internal controls) for comparison. **(D)** Evidence that synergism between DXS/histamine leads to leakage *via* activation of the kallikrein–kinin system was sought in HCP superfused for 10 min with DXS (2 µM) and captopril, followed by 5 min of superfusion with DXS/histamine application (*n* = 9). Prior to the application of histamine, the DXS-treated HCPs were either treated (superfusion interrupted) with the contact phase inhibitor PdSP15a (*n* = 6) or with PKa inhibitor PKSI-527 (*n* = 5) for 5 min. HOE-140 (*n* = 4), the B2R antagonist (B2RA) was applied together with DXS + Captopril. **(E)** For plasma reconstitution experiments, HCPs were superfused with DXS + Captopril for 10 min, followed by topical application (superfusion interrupted) of heparinized hamster plasma for 7 min. Plasma (*n* = 5), and DXS/plasma (*n* = 8). HOE-140 (*n* = 4) was applied along with DXS + Captopril before addition of plasma. Data are expressed as: **(C–E)** Relative fluorescence units (RFUs) (mean ± SD). Statistical analyzes were done by analysis of variance and *t*-test. In **(C)**, *P < 0.05 DXS/Capto/Hist versus DXS/Hist and ^+^*P* < 0.05 for DXS/Hist versus DXS/Crom/Hist and D, *P < 0.05 DXS/PdSP15a/Hist versus DXS/Hist.

Before checking whether *T. cruzi*-induced inflammation involved activation of the MC/KKS pathway, we performed a series of mechanistic studies in a simplified model involving topic application of DXS (500 kDa)—a classical activator of the contact system (53), here used to simulate the effect of MC-derived triggers, such as heparin and polyP, both of which stored in MC granules (11, 12). Prior to the undertaking of IVM studies, we conduced test tube assays to verify whether addition of DXS (20 nM) to citrated hamster plasma activated the contact system, generating enzymatically active PKa. As previously shown with human plasma (42), we found (Figure 1A) that (i) DXS-treated hamster plasma promoted the hydrolysis of the BK flanking sequences of murine kininogen-1 and (ii) the enzymatic cleavage was inhibited by PKSI-527, a well-characterized PKa inhibitor (41).

Next, we checked whether the topically applied DXS (4 µM; added to the superfusate) could elicit plasma leakage (FITC-dextran extravasation) in the HCP *via* activation of the KKS. Intriguingly, DXS did not evoke significant microvascular leakage for at least 30 min (Figure 1B, left panel). Unexpectedly, however, we noticed a subtle extravasation in a few postcapillary venules at later time-points (Figure 1B, left panel), which then rapidly propagated throughout the entire HCP. This delayed plasma leakage elicited by DXS (>30 min) was inhibited both by cromoglycate (Figure 1B, right panel, Figure S1 in Supplementary Material) and by HOE-140, a specific B2RA (Figure S1 in Supplementary Material). These initial results suggested that some extent of MC degranulation occurred after 30 min of HCP exposure to DXS 4 µM. Further downstream, histamine released from MCs could activate endothelial cells and, thus, enable diffusion of plasma-borne contact factors (FXII/PK/HK) through the DXS-laden HCP. Since these effects were blocked by HOE-140, we deduced that BK released at the downstream end of the proteolytic cascade could intensify and spatially propagate inflammation *via* iterative cycles of endothelial B2R activation, plasma leakage and MC degranulation. To test this hypothesis, we next checked whether addition of sub-optimal concentrations of DXS (10-fold lower; 0.4 µM) and histamine (4 µM, likewise added to the superfusate) could act synergistically in the HCP. Indeed, we saw a fourfold increase (*P* < 0.05) in the maximal extravasation of plasma induced by DXS/histamine at 10 min (Figure 1C) as compared to the microvascular leakage evoked by histamine alone (10 min). Moreover, the leakage induced by DXS/histamine was further potentiated by addition of captopril, i.e., inhibitor of BK degradation by ACE; the presence of the ACE inhibitor led to a 12-fold increase in maximal extravasation at 20 min—as compared to histamine alone (10 min, Figure 1C, fold increase calculated after subtraction of the baseline fluorescence). Importantly, the synergistic effects of DXS/histamine were inhibited by (i) cromoglycate, irrespective of absence (Figure 1C) or presence (Figure 1C; Figure S3 in Supplementary Material) of captopril, (ii) HOE-140 (Figure 1D), (iii) the PKa inhibitor PKSI-527 (Figure 1D), and (iv) PdSP15a (Figure 1D), a contact phase inhibitor present in the saliva of the sand fly *Phlebotomus duboscai* (42). To further evaluate whether MC/histamine-driven influx of plasma was the rate-limiting step governing BK release in DXS-HCP, we substituted the histamine “priming” step by adding an aliquot (50 µl) of fresh hamster plasma (heparinized) on top of the HCP. Prior to application on the tissue, we conducted the test tube assay described above (Figure 1C) and confirmed that there was no spontaneous activation of the contact system in the hamster plasma-treated with 4 kDa heparin. Moreover, the presence of heparin 4 kDa did not protect the plasma from PK activation by DXS (Figure S2 in Supplementary Material). As predicted, DXS promptly induced microvascular leakage *via* the kinin/B2R-dependent pathway (Figure 1E). Control experiments revealed that the integrity of the endothelial barrier was preserved in HCP exposed to hamster plasma in the absence of DXS (Figure 1E).

**Figure 2:**
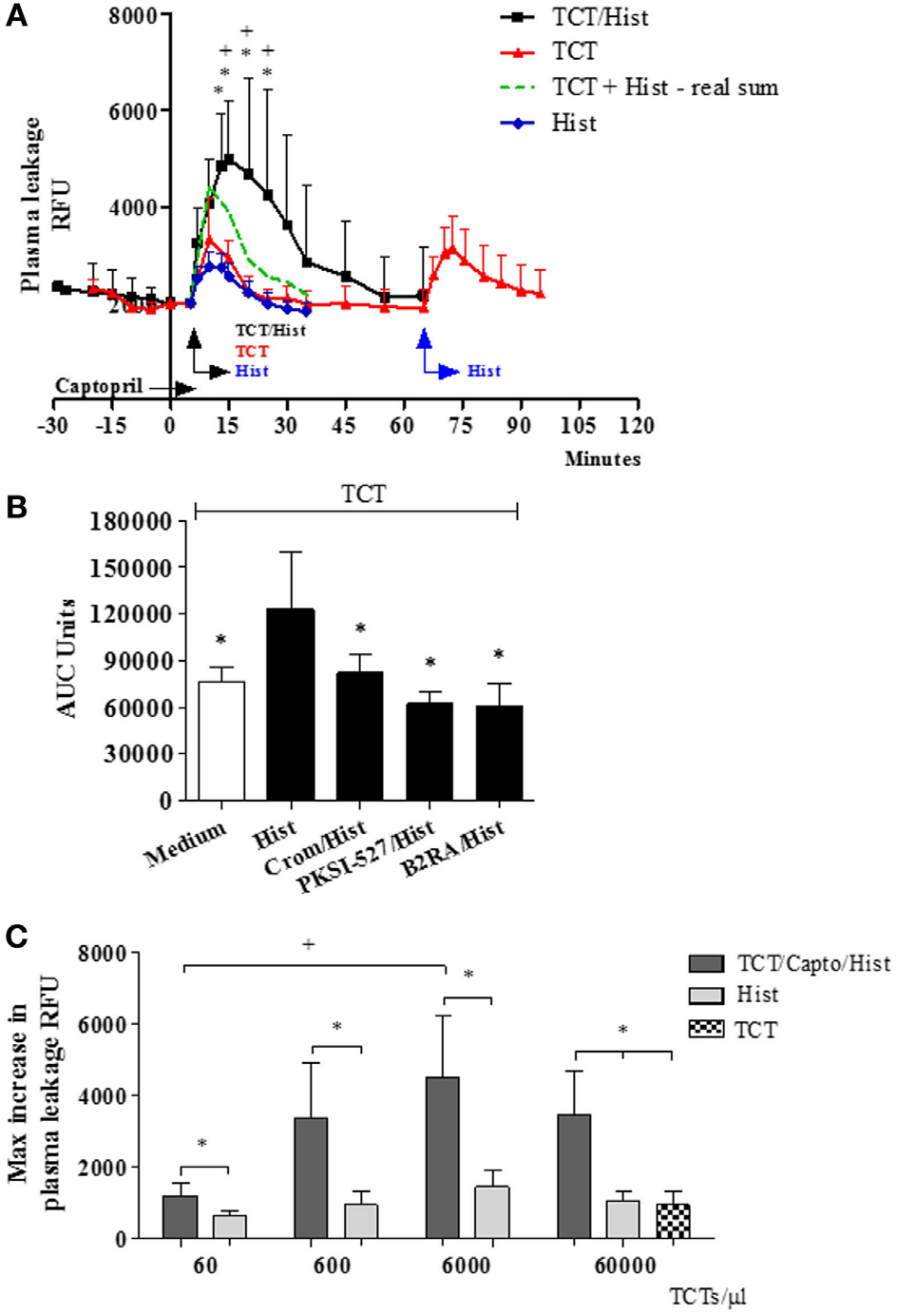
A subtle destabilization of the endothelial barrier potentiates infection-associated inflammation. **(A–C)** Hamster cheek pouches (HCPs) were superfused with captopril for 10 min, followed by application of histamine (4 µM) along with tissue culture trypomastigotes (TCTs) (3 × 10^7^) during 10 min of interrupted superfusion (*n* = 12). TCT control group (*n* = 8) involved HCP treatment with captopril in the absence of histamine. Prior to TCT/histamine application, pharmacological interventions were performed with cromoglycate (pretreatment, *n* = 6); HOE-140 (B2RA; *n* = 5) and PKSI-527 (*n* = 5), applied for 5 min. In A at 60 min after TCT application histamine 4 µM (*n* = 8) was applied as an internal control and these values were then added to the TCT values (real sum – green curve) and used for the calculation of a synergistic effect of histamine and TCT. Results of histamine 4 µM (*n* = 10) applied to HCPs in steady state were inserted as a secondary control. Data are expressed as follows: **(A)** relative fluorescence units (RFUs) over time (mean ± SD), **(B)** area under the curve (AUC ± SD) measured during 30 min. Statistical analysis were done by analysis of variance (ANOVA) and Wilcoxon. In **(A)** *P < 0.05 (TCT/Hist versus TCT) and ^+^*P* < 0.05 (TCT/Hist versus real sum). In **(B)** *P < 0.05 (TCT/Hist versus TCT or TCT/Hist/inhibitor/antagonist) **(C)** HCPs were superfused with captopril for 10 min, followed by topical application of different doses of TCTs (60, 600, 6,000, and 60,000 TCTs/μl) and histamine (*n* = 4) during 10 min of interrupted superfusion (dark gray). At 60 min after TCT application, histamine was applied during 5 min as a control (light gray). Data are expressed as maximal increase in RFU (mean ± SD). Statistical analyzes were done by ANOVA and Wilcoxon (*P < 0.05, TCT/Capto/Hist versus histamine; TCT/Capto/Hist versus TCT; ^+^*P* < 0.05, TCT 60/Capto/Hist versus TCT 6000/Capto/Hist).

In the next series of experiments (Figure 2), the TCTs were topically applied to the HCP jointly with a sub-optimal dose of histamine with the purpose to induce a subtle microvascular leakage in the parasite-laden tissues. As described in previous studies, the superfusion of the HCP is interrupted for a longer period (9 min) to allow for the diffusion/attachment of the motile flagellated parasites to the tissue stroma (see Methods). To minimize ACE/kininase II-dependent degradation of intact kinins during the period of interrupted superfusion, we routinely added the ACE inhibitor captopril to the superfusate (29, 31, 38). As predicted, the application of TCTs to histamine-primed (suboptimal) HCPs induced leakage responses that were far more robust as compared to that induced separately by the pathogen or by histamine (Figure 2A, 328 and 1,094%, respectively, at 15 and 20 min; *P* < 0.014 and 0.037, percentage calculated after subtraction of the baseline fluorescence). The notion that TCTs/histamine acted synergistically at 15, 20, and 25 min (Figure 2A; green versus black squares, *P* < 0.05) was confirmed by analysis of the arithmetic sum of leakage responses from individual hamsters to histamine alone and TCTs alone. Similarly, measurements of area under the curve (up to 30 min) showed that the spatial propagation of inflammation observed in histamine-primed HCPs exposed to TCTs was abolished by: (i) B2RA (HOE-140), (ii) cromoglycate, and (iii) the PKa inhibitor PKSI-527 (Figure 2B, *P* < 0.05). We reached the same conclusion using the peak of macromolecular leakage as the criteria (Figure S4 in Supplementary Material, *P* < 0.05). Control experiments, i.e., performed in the absence of TCTs, showed that plasma leakage induced by histamine alone (4 µM) was not potentiated by captopril, nor was it inhibited by HOE-140, PKSI-527 or cromoglycate (Figure S5 in Supplementary Material). Finally, histamine-sensitized HCPs were topically exposed to decreasing doses of TCTs in order to determine the minimal load of pathogen that was capable of evoking a significant leakage. Our results (Figure 2C) showed that a 100-fold lower load (corresponding to 600 TCTs/μl) still caused a statistically significant increase in plasma leakage (*P* < 0.05) as compared with histamine alone. Collectively, our studies suggest that tissues with leaking postcapillary venules might potentiate the proinflammatory phenotype of trypomastigotes *via* activation of BK-induced feedback loops forged by MC coupling to the KKS.

### *T. cruzi* Elicits Paw Edema *via* Activation of the MC/KKS Pathway

**Figure 3:**
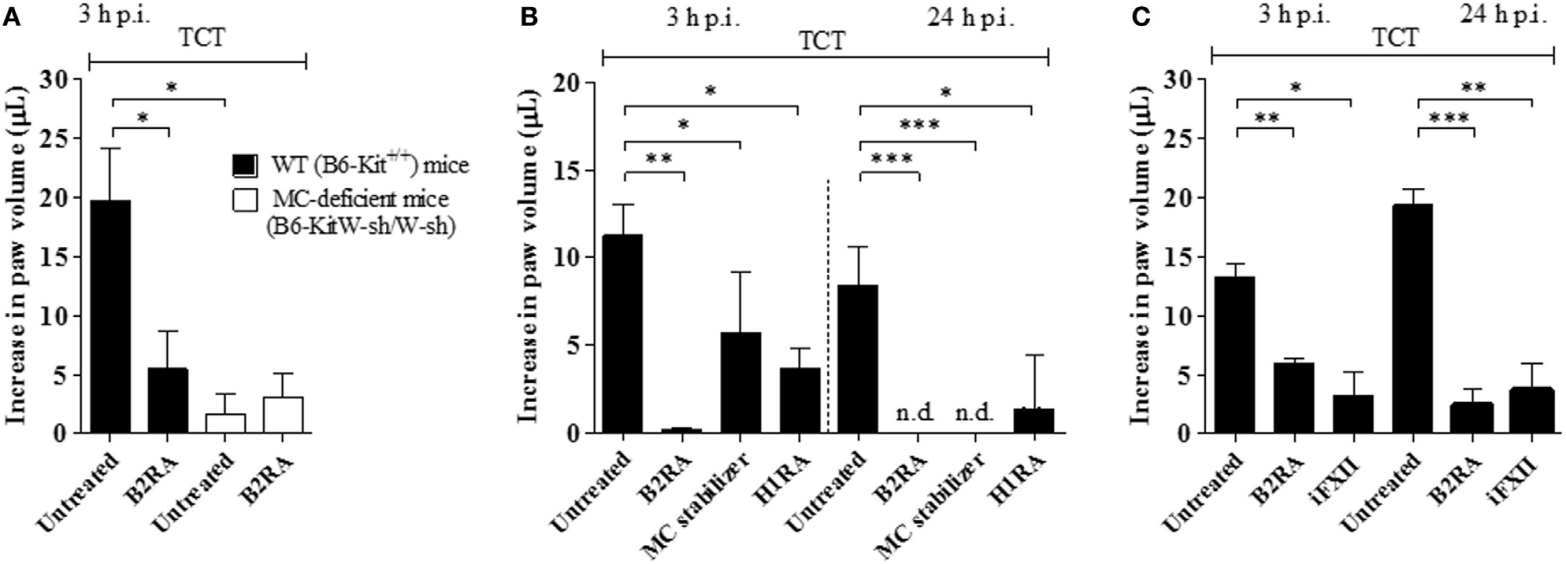
Tissue culture trypomastigotes (TCTs) evoke paw edema *via* the mast cell (MC)/kallikrein–kinin system pathway. **(A)** WT (B6-Kit^+/+^) or MC-deficient mice (B6-kitW-sh/W-sh) were infected with TCTs (10^6^). The contralateral paw received PBS. When indicated, mice were pretreated (−1 h) with the B2R antagonist (B2RA) (HOE-140). **(B,C)** Infected BALB/c mice were pretreated (−1 h) with HOE-140, cromoglycate (MC stabilizer), histamine H1 receptor (H1R) antagonist (mepyramine) or factor XII (FXII) inhibitor (infestin-4). Captopril was used (i.p.) to inhibit kinin degradation. Edema was measured at 3 and 24 h and results are expressed as difference in volume (μl) between infected and contralateral paw (mean ± SD). Data are representative of three independent experiments (five mice/group). Statistical analyzes were done by analysis of variance with Bonferroni post test (*P < 0.05; **P < 0.01, ***P < 0.001).

Although the studies in the HCP provided a framework to investigate the impact of plasma leakage on the proinflammatory phenotype of *T. cruzi*, the anesthetized hamsters must be sacrificed at the end of the IVM experiments (~60 min), thus precluding an analysis of the pathogenic outcome at later time points. Turning to mice models of infection, we next injected Dm28c TCTs in the footpad of B6-KitW-sh/W-sh (MC-deficient) and B6-Kit^+/+^ mice. Edema measurements (3 h) revealed a prominent edema in WT mice (Figure 3A) and a negligible paw response in the mutant strain (Figure 3A). Internal controls in MC-competent mice showed that the edema was inhibited by HOE-140 (B2RA) (72%, *P* < 0.05) (Figure 3A). We next pretreated WT mice with cromoglycate, and found that this classical MC stabilizer inhibited the infection-associated edema (49%, *P* < 0.05) even in animals that received captopril (Figure 3B). Noteworthy, cromoglycate did not inhibit inflammation as efficiently as HOE-140 at this early time point (3 h) (Figure 3B, left panel) but the protective effects of these two drugs equalized at 24 h p.i. (Figure 3B, right panel). Similarly, mepyramine (antagonist of histamine H1 receptor, H1RA) partially inhibited TCT-evoked swelling both at 3 and 24 h p.i. (67%, *P* < 0.05, and 83%, *P* < 0.05, respectively; Figure 3B). To verify whether TCTs evoked B2R-dependent edema *via* MC coupling to the contact system, we next pretreated BALB/c mice with infestin-4, a recombinant protein recently characterized as a specific inhibitor of FXIIa (36). Indeed, infestin-4 drastically inhibited TCT-induced swelling (75%, *P* < 0.05) (Figure 3C), thus further suggesting that activation of the BK/B2R pathway propagates the inflammatory response orchestrated *via* the contact pathway.

### Intracardiac Activation of the MC/KKS Pathway Fuels Heart Parasitism and Infection-Associated Myocarditis/Fibrosis

**Figure 4:**
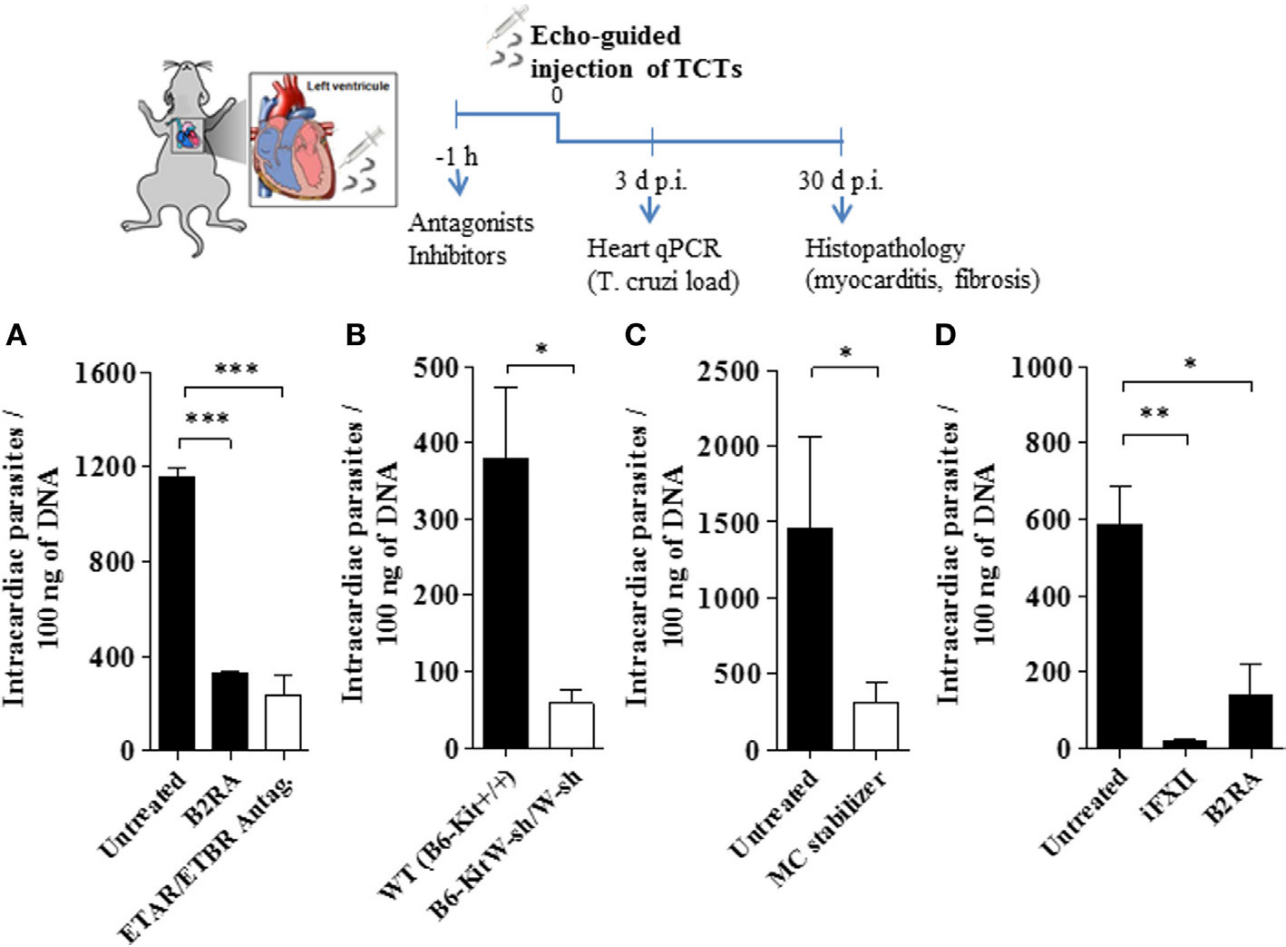
Activation of the kallikrein–kinin system fuels heart parasitism. Measurements of *Trypanosoma cruzi* DNA (3 d.p.i.) in the heart of C57BL/6 infected [10^6^ tissue culture trypomastigotes (TCTs)] intracardiacally **(A,C,D)**, WT (B6-Kit^+/+^) mice or mast cell deficient (B6-kitW-sh/W-sh) mice **(B)**. Where indicated, the mice were pretreated with cromoglycate, B2R antagonist (B2RA) (HOE-140), endothelin A receptor (ET_A_R)/endothelin B receptor (ET_B_R) double antagonist (bosentan) or factor XII (FXII) inhibitor (infestin-4). Results (mean ± SD) of *T. cruzi* DNA by quantitative polymerase chain reaction (qPCR) are representative of two independent experiments (five mice/group). Statistical analyzes were done by analysis of variance with Bonferroni post test (*P < 0.05; **P < 0.01, ***P < 0.001).

Given pieces of evidence that BRs and ETRs serve as gateways for *T. cruzi* invasion of cardiovascular cells *in vitro* (13, 27, 33), here we asked whether intracardiac activation of the MC/KKS pathway could promote parasite infectivity by fostering the proteolytic release of kinins and endothelins, which could then act as infection-promoting signals in heart tissues. Assisted by high-resolution echocardiography, we injected TCTs (Dm28c) directly in the LV of the heart of WT and MC-deficient mice. Heart parasite load (replicating amastigotes) was evaluated 3 d.p.i. by quantifying *T. cruzi* DNA by qPCR. In the second group of experiments, intracardiac challenge with TCTs was performed in WT mice that were pretreated 1 h earlier with (i) cromoglycate, (ii) infestin-4, (iii) HOE-140, and (iv) bosentan (double antagonist of ET_A_R/ET_B_R). Noteworthy, here (unlike the experimental conditions used in the footpad model of infection), the animals were not pretreated with captopril—thus preserving the kinin-degrading function of ACE as well as its competence to convert angiotensin I into the vasoconstrictor angiotensin II in the cardiovascular system. At different time-points (see time-line, Figure 4), we examined extent of heart parasitism (3 d.p.i.) and pathological outcome (histopathology, 30 d.p.i.). Strikingly, we observed that the parasite load in heart tissues declined in WT mice pretreated by HOE-140 (B2RA) (Figures 4A,D; respectively 71 and 76%, *P* < 0.001) or bosentan (ET_A_R/ET_B_R Antag, Figure 4A, 80%, *P* < 0.001). Having established that these GPCR blockers redundantly inhibited intracardiac parasitism in WT mice, we next asked whether cardiac MCs were implicated in this process. First, we compared the levels of parasite DNA in the heart of MC-deficient B6-KitW-sh/W-sh mice versus MC-competent mice. Notably, the parasite load (3 d.p.i.) was sixfold lower in the hearts of the MC-deficient strain as compared to the WT controls (Figure 4B). Given technical difficulties to reconstitute the heart of MC-deficient mice with bone marrow-derived progenitor cells, we re-addressed this question by subjecting WT mice to pharmacological treatment with the MC stabilizer cromoglycate. Consistent with the protective phenotype of MC-deficient mice, we found that heart parasitism (Figure 4C) was sharply reduced in cromoglycate-treated mice (78%, *P* < 0.05). Noteworthy, control experiments performed with cromoglycate (tested *in vitro* at a concentration equivalent to that present in the bloodstream) confirmed that this MC stabilizer did not impair *T. cruzi* infection of primary mouse heart cells (Figure S6 in Supplementary Material). We next pretreated WT mice with the FXIIa inhibitor infestin-4 and verified that the intracardiac levels of *T. cruzi* DNA (Figure 4D; 97%, *P* < 0.01) were sharply decreased.

**Figure 5:**
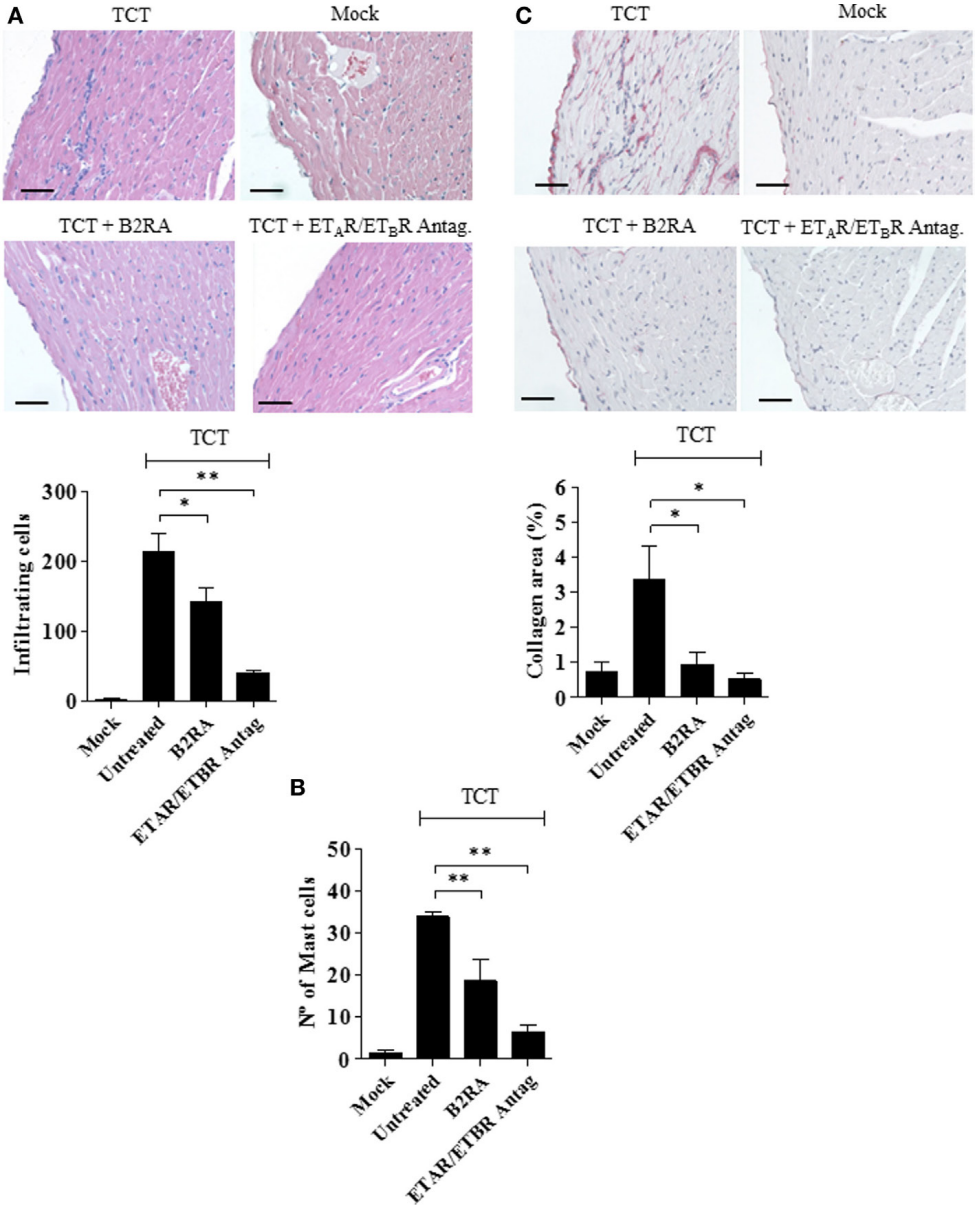
Early targeting of BRs and ETRs inhibit myocarditis/fibrosis. C57BL/6 mice were infected intracardiacally with tissue culture trypomastigotes (TCTs) (10^6^) or PBS (mock). Where indicated, mice were pretreated with B2R antagonist (B2RA) (HOE-140), or endothelin A receptor (ET_A_R)/endothelin B receptor (ET_B_R) antagonist (bosentan). At 30 d.p.i., heart sections were stained with **(A)** hematoxylin and eosin for cellular infiltration, **(B)** Safranin for mast cell detection, or **(C)** Picrosirius Red for collagen deposition. Bars, 50 µm. Data (mean ± SD) are representative of four independent experiments (five mice/group). Statistical analyzes were done by analysis of variance (*P < 0.05; **P < 0.01, ***P < 0.001).

Next, we performed histopathological studies to determine whether the early cardioprotective effects promoted by the GPCR antagonists attenuated heart pathology. Not surprisingly, histopathology performed at 30 d.p.i. revealed that myocarditis/fibrosis was intense in the control infected mice (Figure 5). Interestingly, the elevated infiltration of leukocytes in chagasic mice was accompanied by increased density of intracardiac MCs (Figure 5). Strikingly, we found a significant reduction in the numbers of heart-inflammatory infiltrating leukocytes and of density of intracardiac MCs in *T. cruzi*-infected mice pretreated with B2RA (HOE-140) or ET_A_R/ET_B_R Antag. (bosentan) (Figure 5A, leukocytes; respectively, 34%, *P* < 0.05; and 82%, *P* < 0.001-MC density; respectively, 45%, *P* < 0.01; 80%, *P* < 0.01; Figure 5B). Examination of heart fibrosis at the same time-points again confirmed that these GPCR antagonists were redundantly cardioprotective: the deposition of collagen was markedly attenuated in mice pretreated either with B2RA or with the double ET_A_R/ET_B_R blocker (Figure 5C, respectively 73%, *P* < 0.05; 85%, *P* < 0.05). Collectively, our studies suggest that excessive intracardiac activation of the MC/KKS pathway fueled *T. cruzi* parasitism and worsened myocarditis/fibrosis *via* proteolytic generation of kinins and endothelins.

## Discussion

Standard definitions of immune subversion are built on the assumption that pathogens exploit host cell metabolism and shifts in immunoregulatory responses to survive in hostile environment. Although we have previously demonstrated that activation of the proinflammatory kinin/B2R pathway generates immunoprotective IFN-γ-producing effector T cells against *T. cruzi* (30), here we provide evidence that *T. cruzi* reciprocally benefits from the intracardiac activation of the MC/KKS pathway: acting jointly with endothelins, kinins enhance tissue parasitism *via* activation of B2R.

**Figure 6:**
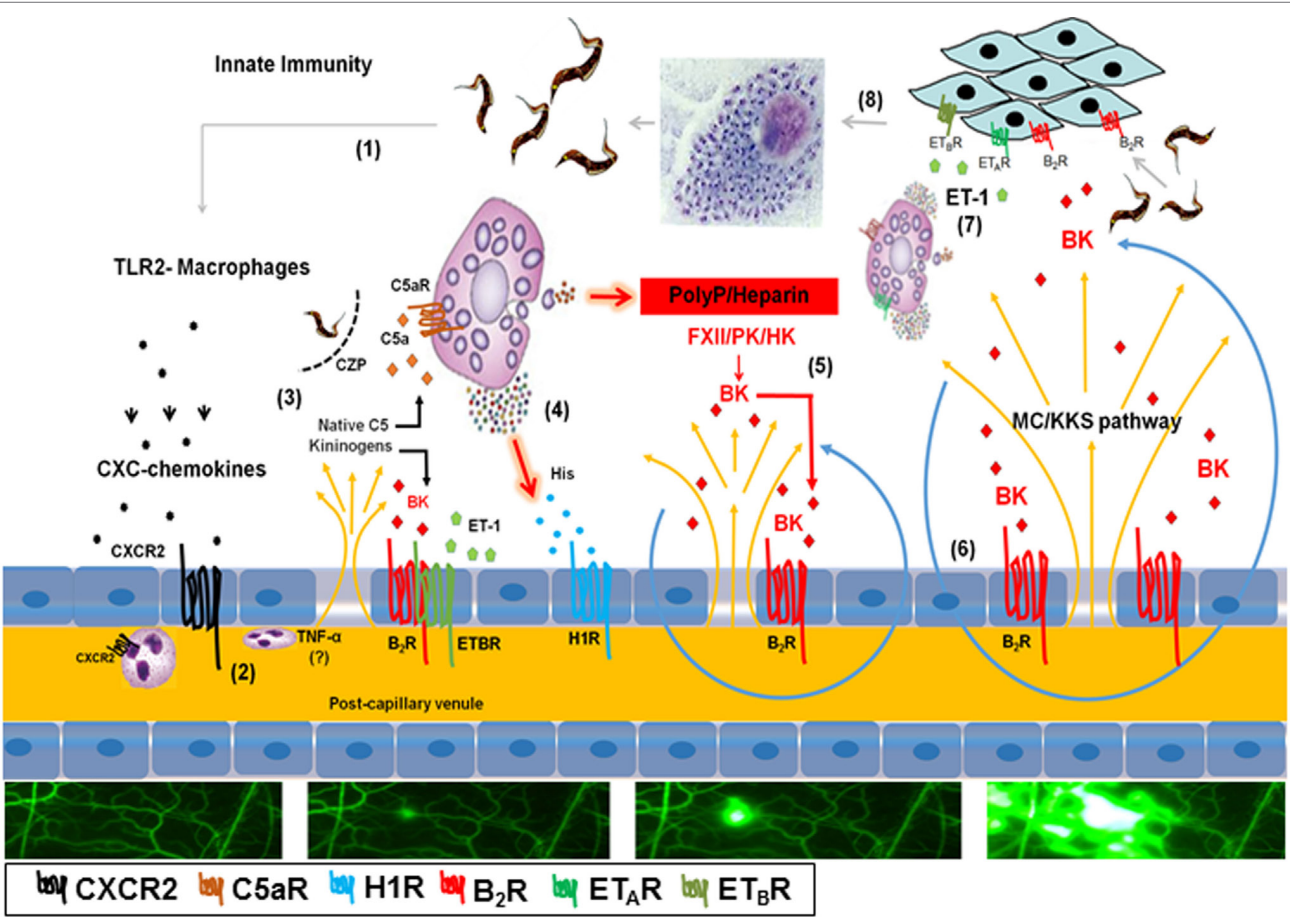
Endothelial barrier breakdown *via* the mast cell (MC)/kallikrein–kinin system (KKS) pathway fuels *Trypanosoma cruzi* infectivity: working hypothesis. (1) Tissue culture trypomastigotes liberated from ruptured pseudocysts initiate inflammation upon sensing by toll-like receptor 2 (TLR2)-expressing tissue macrophages. (2) Secreted CXC chemokines evoke a subtle plasma leakage following CXCR2-dependent activation of endothelium/neutrophils (29, 38); although not explored in this infection model, it has been reported that TNF-α (57) is critically involved in neutrophil-evoked plasma leakage. (3) The diffusion of plasma proteins, including native C5 and kininogens, leads to cruzipain-mediated release of kinins and C5a anaphylatoxin in the parasite-laden tissues (31). (4) Results presented in the current work suggest that cardiac MCs activated by C5a and/or endothelin-1 (not shown in this part of the illustration) release histamine/polyphosphates (PolyP) (contact system activators) from their granules. (5) Activation of histamine H1 receptor (H1R) leads to the exposure of plasma-borne contact factors [factor XII (FXII), high molecular weight kininogen (HK) and plasma prekallikrein] to heparin/PolyP. Further downstream, the activated contact factor plasma kallikrein (PK) releases bradykinin (BK) from HK. (6) BK propagates the leakage response temporally and spatially *via* iterative cycles of endothelial [bradykinin B2 receptor (B2R)] activation (BK/B2R pathway), plasma leakage, MC degranulation, and contact system activation (MC/KKS pathway). (7) Acting jointly with endothelin-1 (ET-1), the released kinins fuel *T. cruzi* infectivity by signaling heart cells that naturally overexpress B2R (13) and ET_A_R/endothelin B receptor (ET_B_R) (33). (8) After endocytic internalization, the flagellated trypomastigotes transform into amastigotes which undergo multiple cycles of binary division in the host cell cytoplasm before transforming into infective trypomastigotes.

Since chronic Chagas disease last several years, occasional fluctuations in immunoregulation may favor intracellular parasite outgrowth in scattered heart cells. Sporadically, a few heavily parasitized host cells rupture, swarming the surrounding tissues with infective trypomastigotes. Within minutes, the endothelial barrier might open in response to endogenous alarmins and/or proinflammatory molecules shed from the trypomastigote surface (Figure 6). It is still unclear whether the genetic diversification of the *T. cruzi* species contributes to the clinical pleiomorphism of CCC (54). Of interest in this context, the expression levels of proinflammatory galactosyl-bearing lipid anchors (55) vary within the genetically diversified *T. cruzi* species. In previous studies performed with the Dm28c strain (29, 31, 33, 38), we demonstrated that Dm28c TCTs (but not avirulent Dm28c epimastigotes) evoke a subtle endothelial barrier destabilization *via* a trans-cellular activation pathway (TLR2/CXCR2/ET_A_R/ET_B_R/B2R/C5aR) initiated by tGPI (TLR2 ligand) and amplified by the kinin-releasing activity of cruzipain (see scheme on Figure 6). In this paper, we showed evidence that the trans-endothelial diffusion of plasma—here sought through the activation of the MC/KKS pathway—enhances both the proinflammatory phenotype and infectivity of Dm28c TCTs. In order to simulate tissues irrigated by “leaky” microvessels, we added sub-optimal doses of histamine jointly with the topically applied TCTs. As predicted, the microvascular leakage response evoked by TCTs was potentiated by BK-induced inflammatory cascades coordinated *via* the MC/KKS pathway. Although the roles of MC-derived polyP/heparin were not directly investigated here, the mechanistic insight that inspired this study came from simplified model studies performed with DXS, a potent activator of the contact system (53). Unexpectedly, the topically applied DXS did not induce any microvascular alterations in steady-state HCP for at least 30 min; thereafter, however, a few localized “leaks” appeared, triggering a MC/KKS (contact phase) driven inflammatory response that rapidly spread throughout the HCP. Combined, the IVM studies with DXS and TCTs support a linear model of BK-induced opening of the endothelial barrier initiated by a subtle influx of plasma contact factors. Assays performed with different contact phase inhibitors suggest that BK, once liberated by PKa, triggers iterative cycles of endothelial (B2R) activation, plasma leakage, MC degranulation, and PolyP/heparin-induced activation of contact factors (Figure 6).

In the second part of this paper, we studied the pathogenic role of the MC/KKS pathway using two different mouse models of infection. Congruent with the IVM data generated in the HCP, we first showed that TCTs induced footpad edema *via* activation of BK/B2R-inflammatory cascades (orchestrated *via* the MC/KKS pathway). Extending these studies to the cardiac settings, we next inoculated TCTs in the heart of WT or MC-deficient mice and measured *T. cruzi* DNA levels at 3 d.p.i. These experiments revealed that intracardiac parasitism was markedly reduced in MC-deficient mice or, alternatively, in cardiac tissues of WT mice pretreated either with (i) cromoglycate (ii) infestin-4 (iii) HOE-140 or (iv) bosentan (non-selective ET_A_R/ET_B_R antagonist). Based on these findings, we inferred that targeting of the MC/KKS pathway stabilized the endothelial barrier, hence ultimately reducing the levels of infection-promoting signals (kinins and endothelins) generated in the parasite-laden heart tissues (Figure 6). It remains to be determined whether the GPCR blockers HOE-140 and bosentan inhibited parasite infectivity by acting as endothelial barrier stabilizers, and/or acted further downstream, directly blocking GPCR-mediated uptake of TCTs (Figure 6) as originally documented in culture systems (13, 27, 32, 33). Another possibility is that *T. cruzi* infection is fueled by ROS (56), presumably reflecting synergism between innate immunity (TLR2/CXCR2 pathway) (38) and the short-lived proinflammatory polypeptides generated further downstream, such as kinins and ET-1. The latter hypothesis is consistent with the view that plasma membrane lesions evoked by ROS (fueled by kinins and endothelins) might trigger a house-keeping mechanism of membrane repair (Ca^2+^/acid sphingomyelinase/ceramide pathway) that drives *T. cruzi* internalization into ceramide-rich endocytic vesicles—as originally proposed by Ref. (45).

Although not addressed here, it will be interesting to determine whether parasitized heart cells in our intracardiac model of infection upregulate ET-1 expression in our controls (i.e., in the absence of pharmacological intervention on the MC/KKS pathway) as documented in conventional models of Chagas heart disease (26). If confirmed, it will be necessary to evaluate whether ET-1 secretion in *T. cruzi*-infected heart tissues depends on the innate recognition of intracellular parasites (amastigotes). In principle, this mechanism may either involve (i) TLR9, recently defined as a sensor of unmethylated CpG motifs of *T. cruzi* DNA (58) (ii) TLR7, activated by parasite-derived RNA (59) and/or activation of NLRP3 inflammassome (60).

Lessons coming from studies of innate immunity in models of heart injury induced by pressure overload (61) might provide clues to understand the mechanism underlying the cardioprotective effects of GPCR blockers documented in our study. Using DNAse II-deficient mice, the above-mentioned authors verified that, under these stressful conditions, the excessive accumulation of host mitochondrial DNA (symbiont-like) in autolysosomes upregulates inflammation *via* activation of TLR9. In principle, it is possible that TLR9-dependent sensing of *T. cruzi* DNA (amastigotes) might upregulate ET-1 secretion. In mice pretreated with HOE-140 or bosentan, the intracardiac load of *T. cruzi* parasites was markedly reduced. Thus, it is conceivable that the pharmacological targeting of the MC/KKS pathway reduced intracardiac inflammation and tissue parasitism to levels that are beyond the threshold required to upregulate ET-1 *via* activation of innate receptors. Consistent with this viewpoint, studies of MC function in other models of heart inflammatory diseases appointed cardiac MCs as central player in cardiac remodeling (62). Our findings that the density of intracardiac MCs (30 d.p.i.) was increased in mice subjected to intracardiac challenge with *T. cruzi* is congruent with clinical evidence showing that (i) MC density is increased in heart tissues from HIV-reactivated CCC patients (63). Although our intracardiac model of (acute) infection is admittedly artificial, it was surprising that severity of myocarditis and heart fibrosis in WT mice (evaluated at 30 d.p.i.) was blunted in animals that were pretreated either with B2R or ET_A_R/ET_B_R antagonists. Intriguingly, the density of cardiac MCs was also reduced in WT mice that were subjected to the single-dose treatment with these GPCR blockers at the time of intracardiac challenge. Combined, these results suggest that endothelins/kinins, acting jointly, might upregulate trans-endothelial migration of MC precursors and/or enhance MC maturation following recruitment to the parasitized heart tissues. Future studies may clarify whether cardiac MCs activated by ET-1 may interconnect intracellular innate responses (orchestrated by TLRs and/or inflammassome) to the proteolytic circuitry that propagates inflammation and heart fibrosis *via* the KKS. While these pathogenic processes are in march in chagasic patients, it is possible that the sporadic formation of inflammatory edema may provide *T. cruzi* trypomastigotes with an opportunity to invade heart cells that naturally overexpress bradykinin and endothelin receptors. In summary, by studying the impact infection-associated microvascular leakage on the pathogenesis of *T. cruzi* infection, our study provides a new mechanistic framework to investigate the role of proteolytic networks, such as the KKS, on host/parasite balance in Chagas heart disease.

## Ethics Statement

All animal care and experimental procedures were performed in accordance to the Brazilian guidelines (Brazilian Directive for Care and Use of animals for Teaching and Research—DBCA) published by the Brazilian Council for Control of Animal Experimentation (Conselho Nacional de Controle de Experimentação Animal—CONCEA, http://www.mct.gov.br/upd_blob/0234/234054.pdf) and Federal Law 11.794 (October 8, 2008). Studies involving animals are reported in compliance with the ARRIVE guidelines (27, 28). The protocols used in this study were approved by the Institutional Ethical Committee of Federal University of Rio de Janeiro (code number: IBCCF 101, and license protocol number 014).

## Author Contributions

CN, DA, AO, and ES designed and performed experiments, analyzed, and interpreted data. CC-P conducted histopathological analysis of heart tissues. LV performed enzymatic essays. GB performed echo-guided intracardiac injection. ER and JM performed and analyzed heart parasitism. RS and LA performed animal experimentation. CN, DA, RS, LV, AC, AO, and ES also helped with the writing and edition of the manuscript. MA, MCS, LJ, PA, and AC contributed providing research tools. FS performed statistical analysis. JS conceived the study and wrote the manuscript.

## Conflict of Interest Statement

The authors declare that the research was conducted in the absence of any commercial or financial relationships that could be construed as a potential conflict of interest.

## Abbreviations

BK, bradykinin; B1R, bradykinin B1 receptor; B2R, bradykinin B2 receptor; HK, high molecular weight kininogen; LK, low molecular weight kininogen; LBK, lysyl-BK; GPCR, G protein-coupled receptor; ACE, angiotensin-converting enzyme; PKa, plasma kallikrein; KKS, kallikrein–kinin system; FXII, coagulation factor XII; MCs, mast cells; CCC, chronic chagasic cardiomyopathy; HCP, hamster cheek pouch; ET-1, endothelin-1; ET_A_R, endothelin A receptor; ET_B_R, endothelin B receptor; DXS, dextran sulfate; TCTs, tissue culture trypomastigotes; H1R, histamine H1 receptor; polyP, polyphosphates.
