# Supplementary material for: Mast Cell Coupling to the Kallikrein–Kinin System Fuels Intracardiac Parasitism and Worsens Heart Pathology in Experimental Chagas Disease
Source: Front Immunol. 2017 Aug 2;8:840. doi: 10.3389/fimmu.2017.00840 (PMC5539176; doi:10.3389/fimmu.2017.00840)

Supplementary Material

Mast Cell Coupling to the Kallikrein-kinin System Fuels Intracardiac Parasitism and Worsens Heart Pathology in Experimental Chagas Disease

Clarissa R. Nascimento[1†], Daniele Andrade[1†], Carla Eponina Carvalho-Pinto[2], Rafaela Rangel Serra[1], Lucas Vellasco[1], Guilherme Brasil[1], Erivan Schnaider Ramos-Junior[1,3], Julia Barbalho da Mota[1], Larissa Nogueira Almeida[1], Marcus V. Andrade[4, 5], Maria de Nazaré Correia Soeiro[6], Luiz Juliano[7], Patrícia Hessab Alvarenga[8], Ana Carolina Oliveira[1], Fernando Lencastre Sicuro[9], Antônio C. Campos de Carvalho[1], Erik Svensjö[1] and Julio Scharfstein[1*]

***Correspondence:**
Julio Scharfstein.

jscharf2@gmail.com

Supplementary Figure 1. The delayed leakage (>30 min) induced by DXS is abolished upon treatment with MC stabilizer or B2R antagonist. Maximal Leakage responses (RFU, mean ± SD) recorded in the HCP sensitized for at least 30 min with DXS (4 µM). The graph depicts responses induced by DXS in controls (absence of treatment) or HCP pretreated with cromoglycate (40 mg/kg, i.p., n=2) and HOE-140.

Supplementary Figure 2. Enzyme assays showing that heparinized hamster plasma is not spontaneously activated. Assay was performed with HK mimetic substrate detect PKa activity in fresh hamster plasma (heparin 4 kDa) incubated with the contact activator DXS (4 nM). The kinetics

of hydrolysis was performed at 37°C. Substrate hydrolysis was monitored by the increase of fluorescence with time (mean ± SD). Data are representative of 3 independent experiments run in duplicates.

Supplementary Figure 3. Microvascular leakage in the HCP sensitized with DXS and captopril is abolished upon treatment with MC stabilizer. HCPs were superfused for 10 min with DXS (2 µM) and captopril, followed by 5 min of histamine (4 µM) application. Where indicated, the hamsters were pretreated (i.p.) with cromoglycate. Data show 3 independent experiments.

Supplementary Figure 4. The microvascular leakage induced by TCT/histamine involves the MC/KKS pathway. TCT control group (n=8) involved HCP treatment with captopril in the absence of histamine. Prior to TCT/histamine application, pharmacological interventions were performed with cromoglycate (40 mg/kg i.p.,pretreatment, n = 6); HOE-140 (n=5) and PKSI-527 (n=5), applied for 5 min. Data are expressed as maximal RFU (mean ± SD), measured during 30 min. Statistical analysis were done by ANOVA and Wilcoxon. * $P < 0.05$ (TCT/Hist versus TCT or TCT/Hist/ inhibitor/antagonist).

Supplementary Figure 5. Specificity controls showing that Captopril, HOE-140 and cromoglycate do not modulate plasma leakage induced by histamine. Maximal leakage responses (Mean ± SD) induced by histamine 4 µM during 5 minutes of superfusion of HCP (steady state). Histamine was applied in HCP pretreated (or not, control) with captopril (10 µM, n= 9), cromoglycate (40 mg/kg i.p., pretreatment, n=6), (4) PKSI-527 (20 µM, n=5) and (5) HOE-140 (0.5 µM, n = 6). There is no statistical statistic difference between the histamine responses ($P > 0.05$).

Supplementary Figure 6. The MC stabilizer cromoglycate does not impair *T. cruzi* infection of primary heart cells. Invasion assays performed by incubating primary heart cells with Dm28c TCTs (parasite/host ratio 1:1) in BSA-DMEM supplemented, or not, with MC stabilizer (cromoglycate). HOE-140 was added as an internal control. After 3 h of interaction, the host cells were stained with Giemsa. Representative results of 2 or 3 independent experiments were expressed as number of intracellular parasites/100 cells (mean ± SD).

Supplementary Figure 1

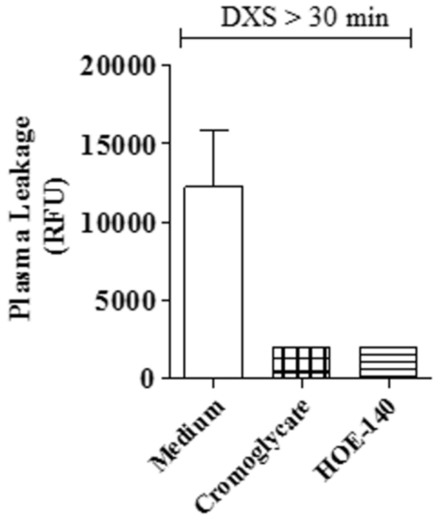

Supplementary Figure 2

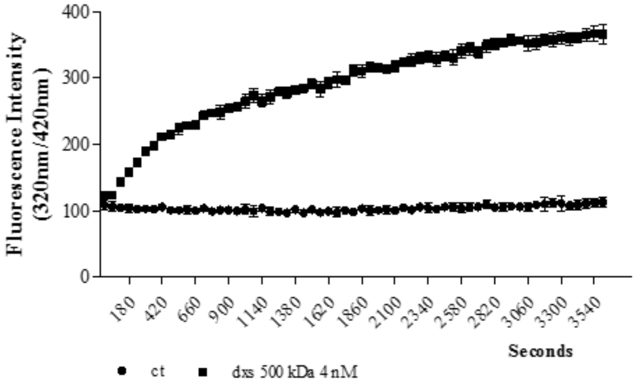

Supplementary Figure 3

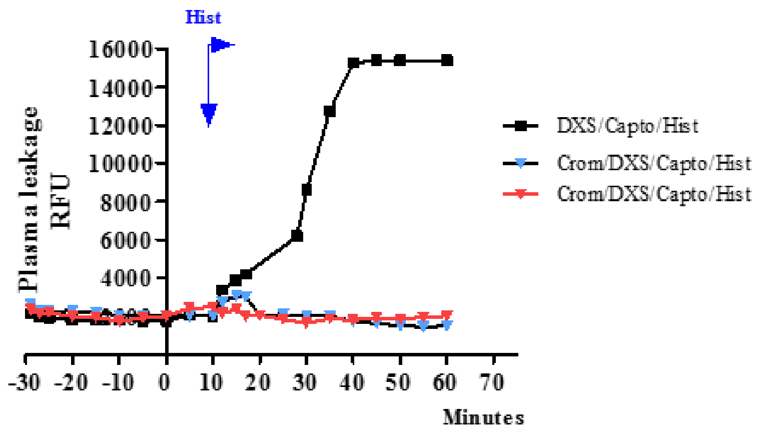

Supplementary Figure 4

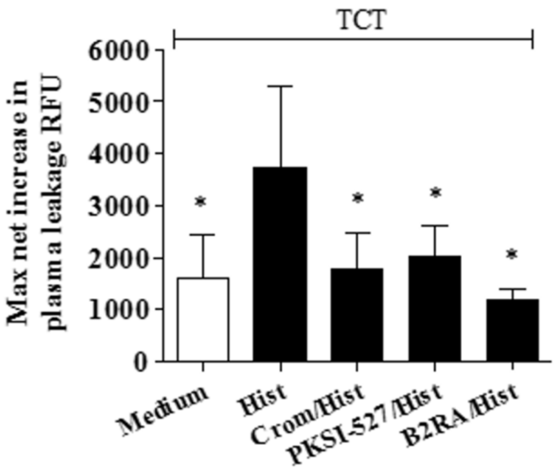

Supplementary Figure 5

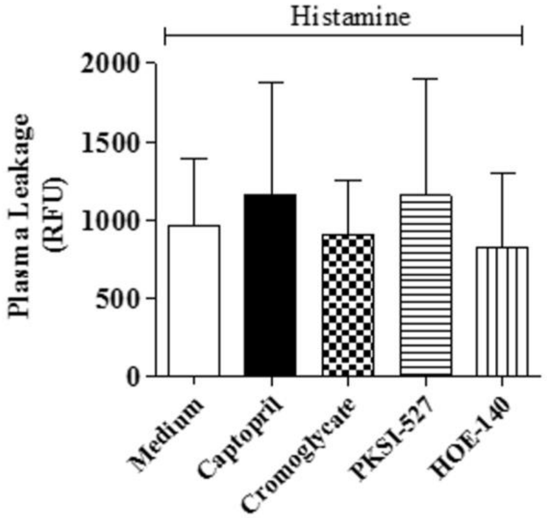

Supplementary Figure 6

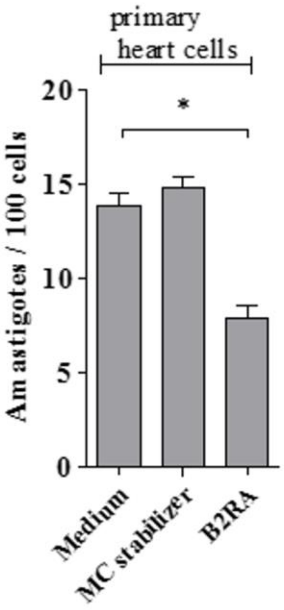

Supplement: Supplementary file 1 [file Presentation_1.pdf]
